# Supplementary material for: SARS-CoV-2 mucosal antibody development and persistence and their relation to viral load and COVID-19 symptoms
Source: Nat Commun. 2021 Sep 23;12:5621. doi: 10.1038/s41467-021-25949-x (PMC8460778; doi:10.1038/s41467-021-25949-x)
Supplement: Supplementary file 1 — Supplementary Information [file 41467_2021_25949_MOESM1_ESM.pdf]

SARS-CoV-2 mucosal antibody development and persistance and their relation to viral load and COVID-19 symptoms

Supplementary material

Graphical abstract

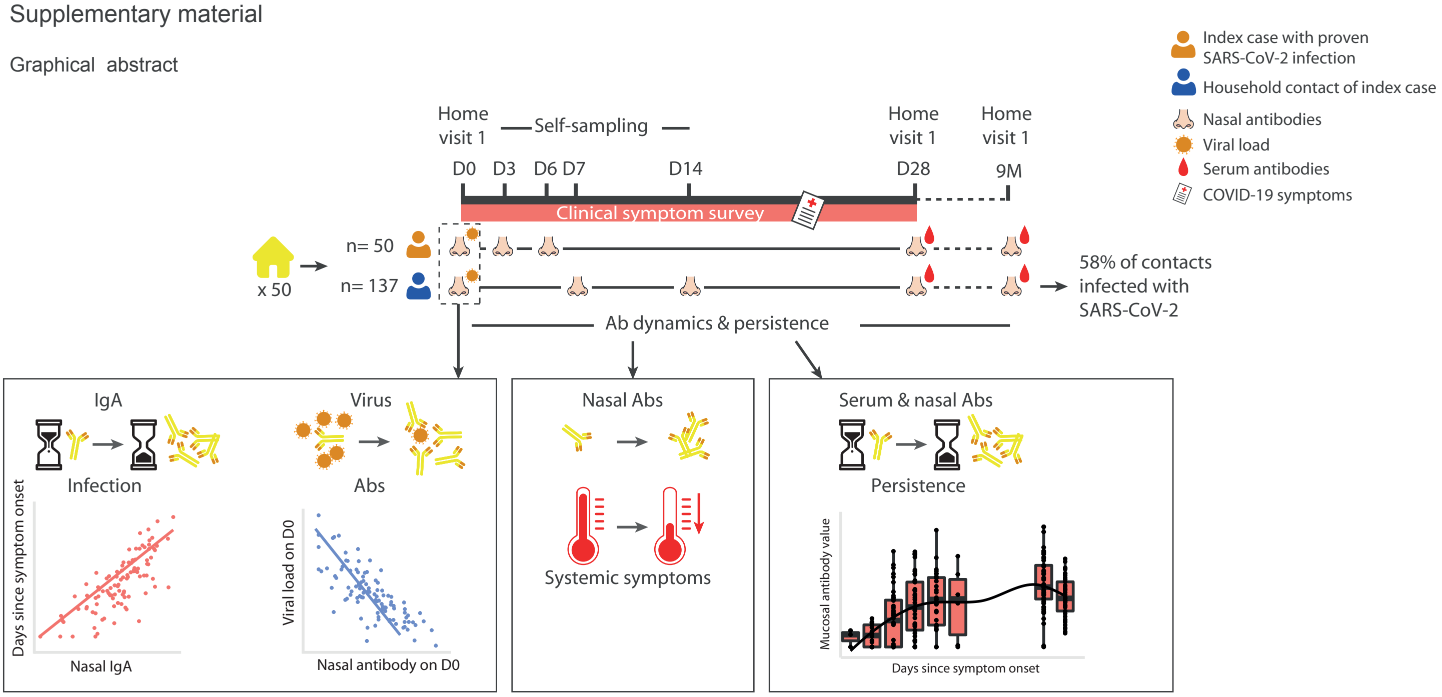

Figure S1

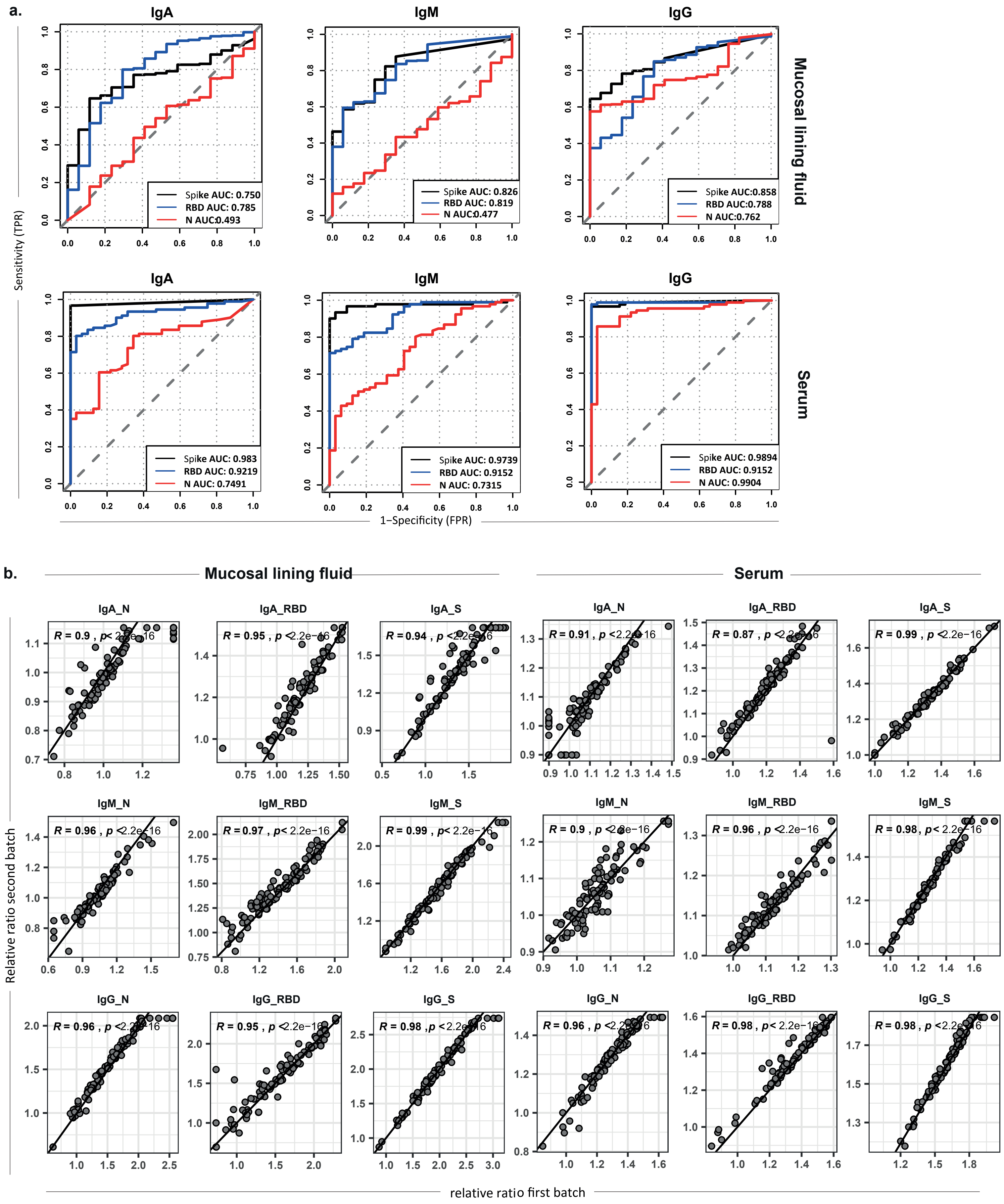

Figure S1. Validation of the multiplex immuno-assay (MIA) performance. a.) Receiver operating characteristic (ROC) curves were produced for serum and mucosal lining fluid for each antigen antibody combination. Pre-corona cohort samples were used as a negative control (MLF N=17, serum N= 32) and PCR positive cases were used as the positive control (MLF N= 464*, serum N=92). Areas under the curve (AUCs) are depicted in the figure for each antigen (N= red, RBD=blue and S=black). b.) Reproducibility of MIA assay was assessed by comparing two batches, which were analysed approximately 9 months apart. Two-sided Spearman correlations were calculated and are depicted in the graph, together with a x=y line and a p-value (all correlations had a p-value < 2.2*1016). Antibody levels are expressed as a ratio compared to the mean of pre-corona samples. * all MLF timepoints were used from the PCR positive participants. Source data are provided as a Source Data file.

Figure S2

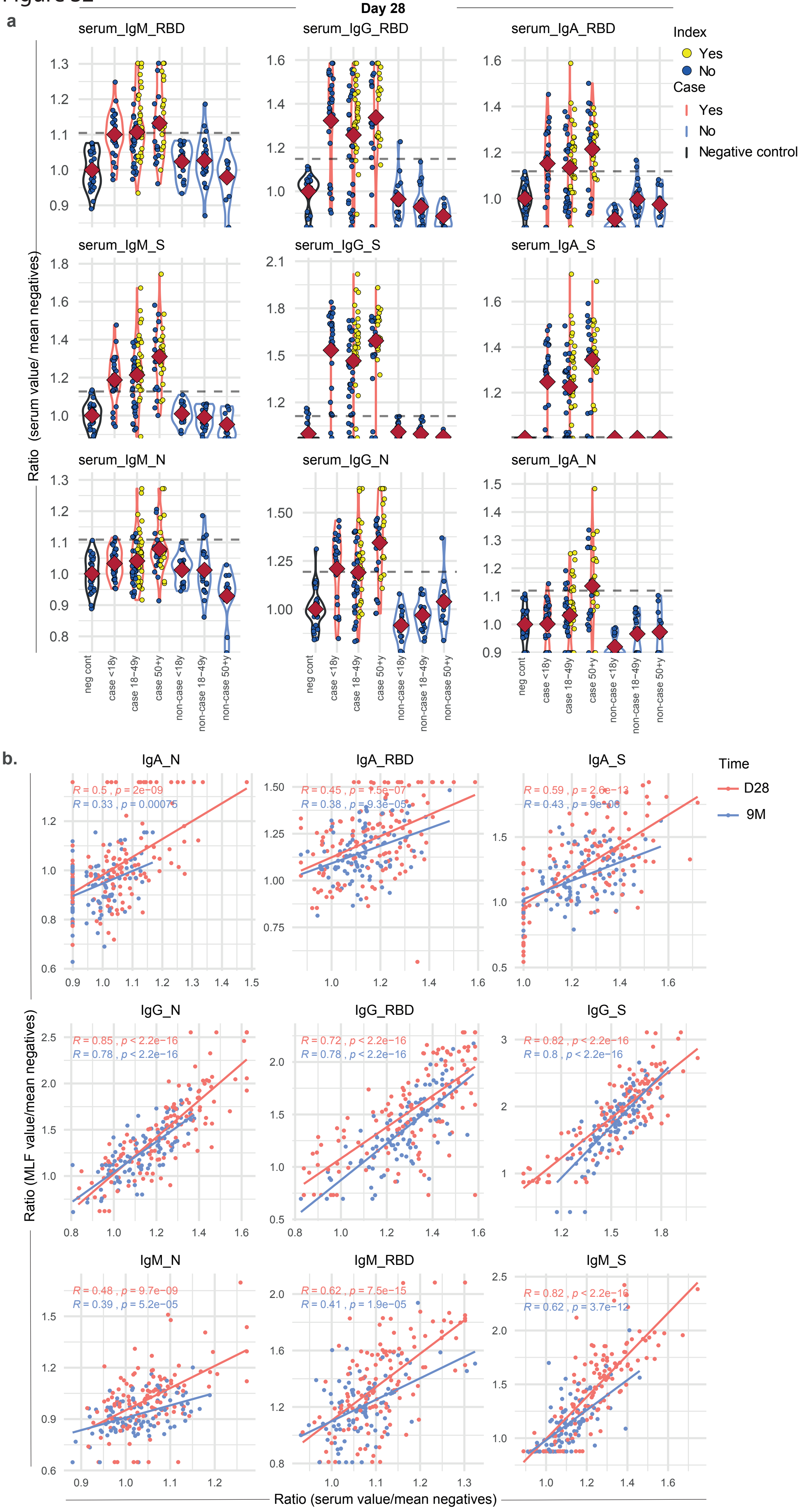

Figure S2. Serum antibody levels against SARS-CoV-2 in COVID-19 cases and uninfected household members. a.) IgM, IgG, and IgA serum antibody responses against SARS-CoV-2 spike protein (S), receptor binding domain (RBD), or nucleocapsid (N). Data are shown for pre-pandemic control samples (N=32, black violin plot). cases (N=130, red violin plots) or non-cases (N=57, blue violin plots). Violin plots display the mean value (red diamond), the range, and the individual data points (yellow=index cases, blue=household contacts). Antibody levels are expressed as a ratio compared to the mean of the pre-pandemic controls, and a threshold for seroconversion (dashed line) was defined by the mean + 2*sd of the control samples. b.) Serum antibody measures against SARS-CoV-2 for each antibody/antigen combination were correlated with the mucosal antibody levels, for day 28 (red line) and nine months (blue line). Antibody levels are expressed as a ratio compared to the mean of the pre-pandemic controls. Data are shown for all cases (N=129 day 28, N=100 nine months). Two-sided Spearman correlations were calculated and p-values are reported in the figure. Source data are provided as a Source Data file.

Figure S3

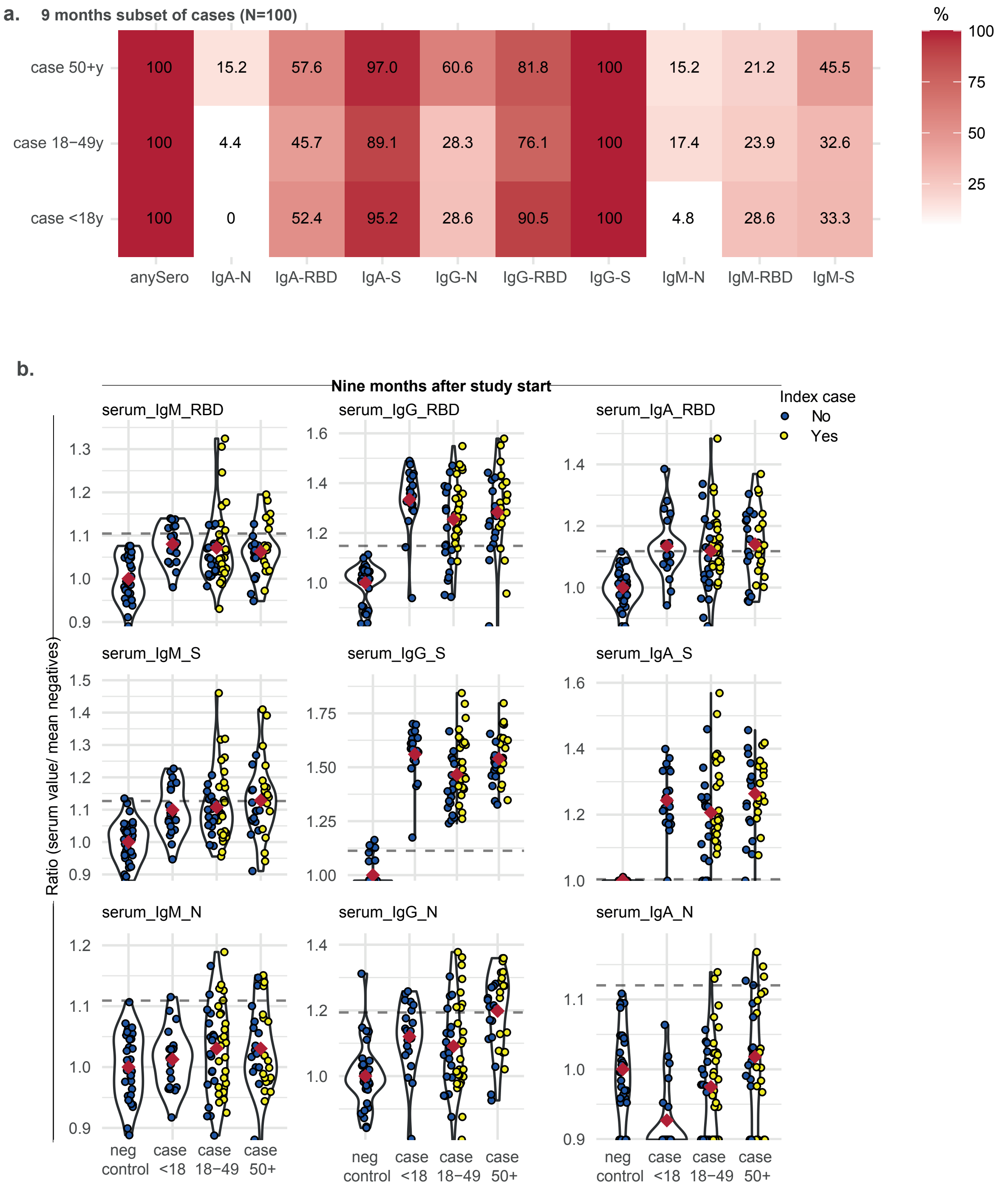

Figure S3. Serum antibody levels persist up to nine months after SARS-CoV-2 infection. a.) Seroconversion heatmap of cases at nine months (N=100), for each antibody/antigen combination, as well as a seroconversion rate for any single antibody measurement (anySero), split into age groups. Seroconversion was defined by the mean + 2*sd of the pre-pandemic control samples. b.) Serum antibody levels of cases at nine months after study start, for each antibody/antigen combination. Seroconversion level is shown by the dashed line. Violin plots display the mean value (red diamond), the range, and the individual data points (yellow=index cases, blue=household contacts). Source data are provided as a Source Data file.

Figure S4

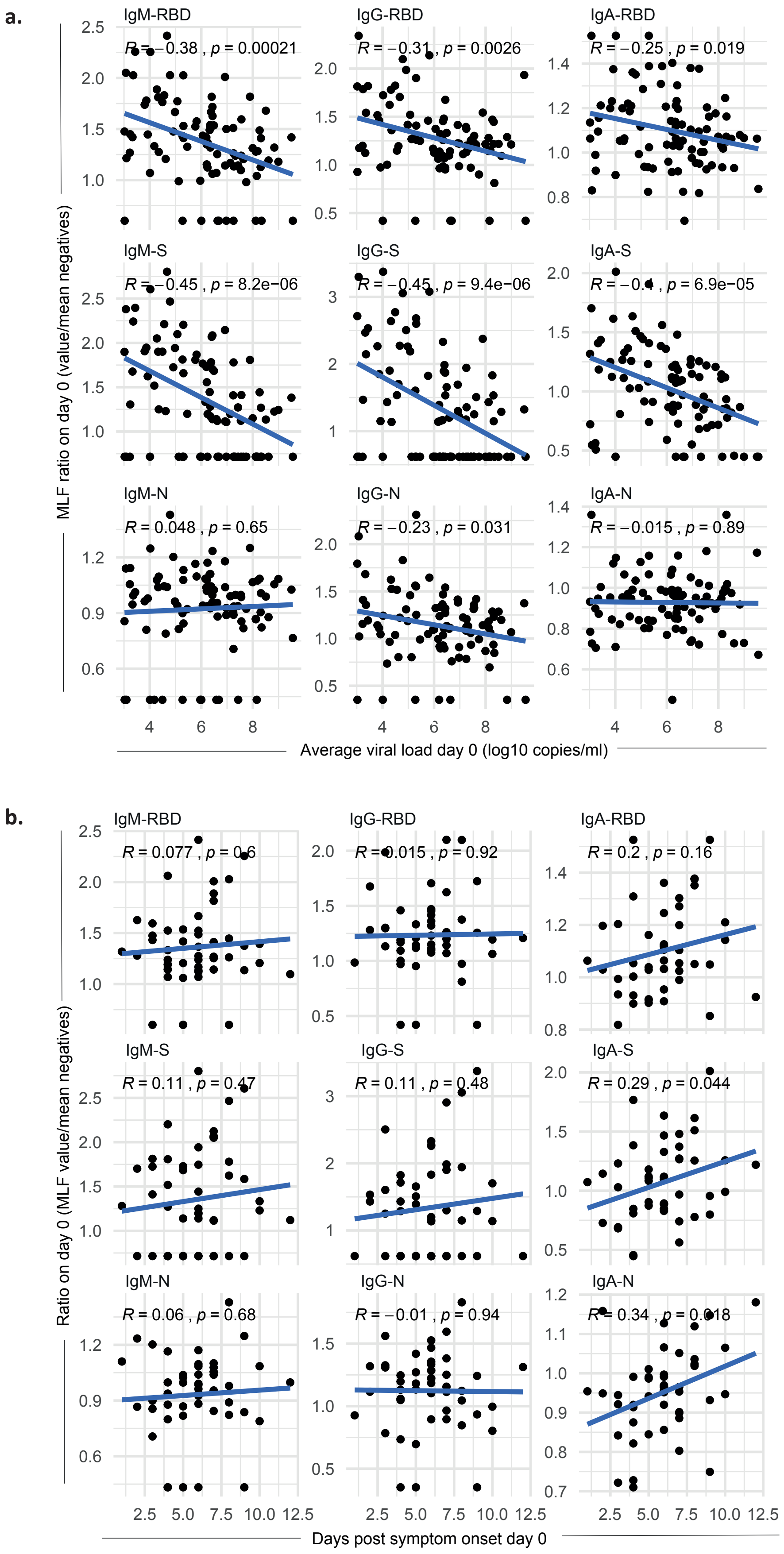

Figure S4. Mucosal antibody responses correlate with viral load and day of symptom onset. a.) IgM, IgG and IgA antibody responses against SARS-CoV-2 spike (S), receptor binding domain (RBD) and nucleocapsid (N) at study start were correlated with viral load at study start, for all PCR positive cases (N=94). Two-sided Spearman correlations and p-values are depicted in the figure b.) mucosal antibody levels of antibody/antigen combinations mentioned in a) were correlated with the days post symptom onset at study start, for all index cases (N=49). Two-sided Spearman correlations and p-values are depicted in the figure. Source data are provided as a Source Data file.

Figure S5

a.

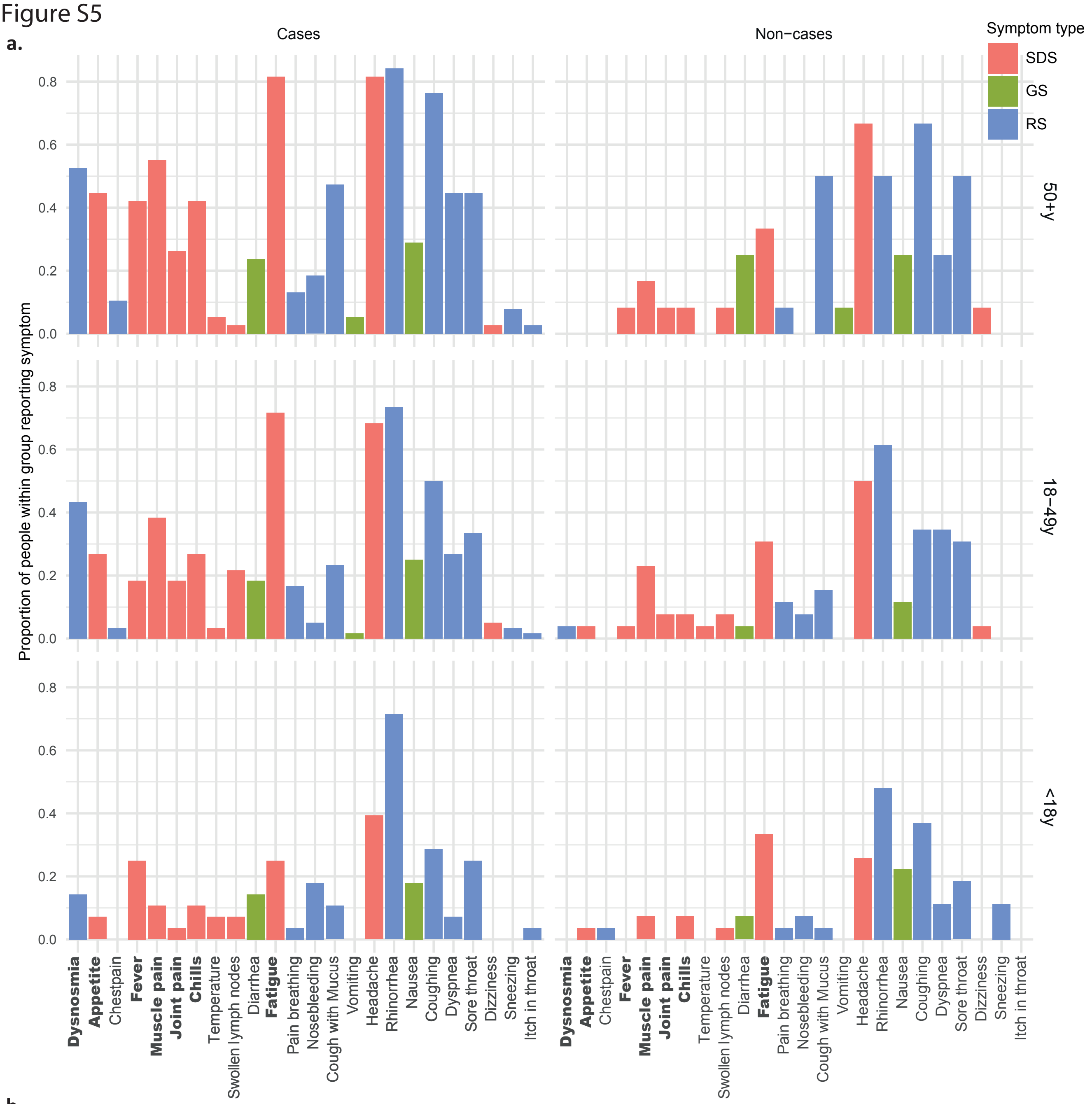

b.

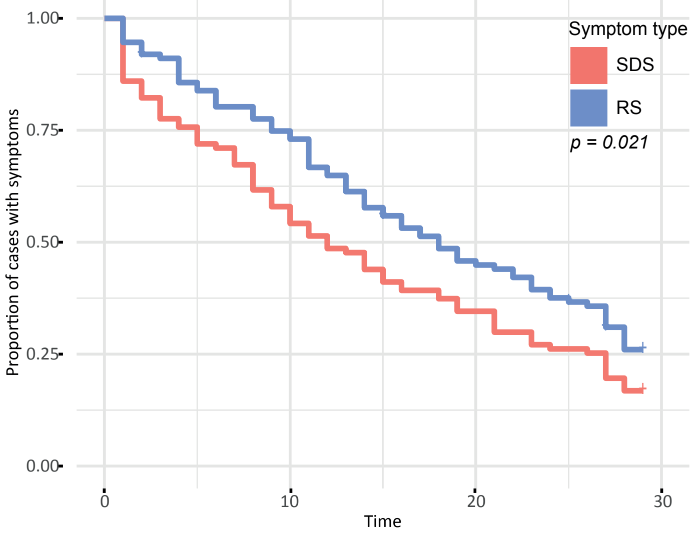

Figure S5. Clinical presentation of mild COVID-19 disease. a.) All participants (N=186) completed a daily symptom survey covering 23 symptoms for 28 days during the study period. The number of participants that reported a given symptom at any time during the study period is presented per age group, split into cases and non-cases. Symptoms were categorized into either respiratory symptoms (RS, blue), systemic disease symptoms (SDS, red), or gastrointestinal symptoms (GS, green). Symptoms are sorted on the change between cases and non-cases, with the more case-specific symptoms on the left, and the general symptoms on the right of the graph. Symptoms that were significantly more often reported by the cases as defined using the two-sided Fisher's exact test are bolded. Headache and rhinorrhea were borderline significant (p=0.07 and 0.11, respectively) b.) Symptom durations in cases (N=130) were calculated for RS and SDS, and compared using a Kaplan Meier analysis. The probability of becoming symptom-free at a given time post-symptom onset is depicted. The two-sided log-rank p-value represents the statistical difference between resolution of SDS and RS. Source data are provided as a Source Data file.

Figure S6

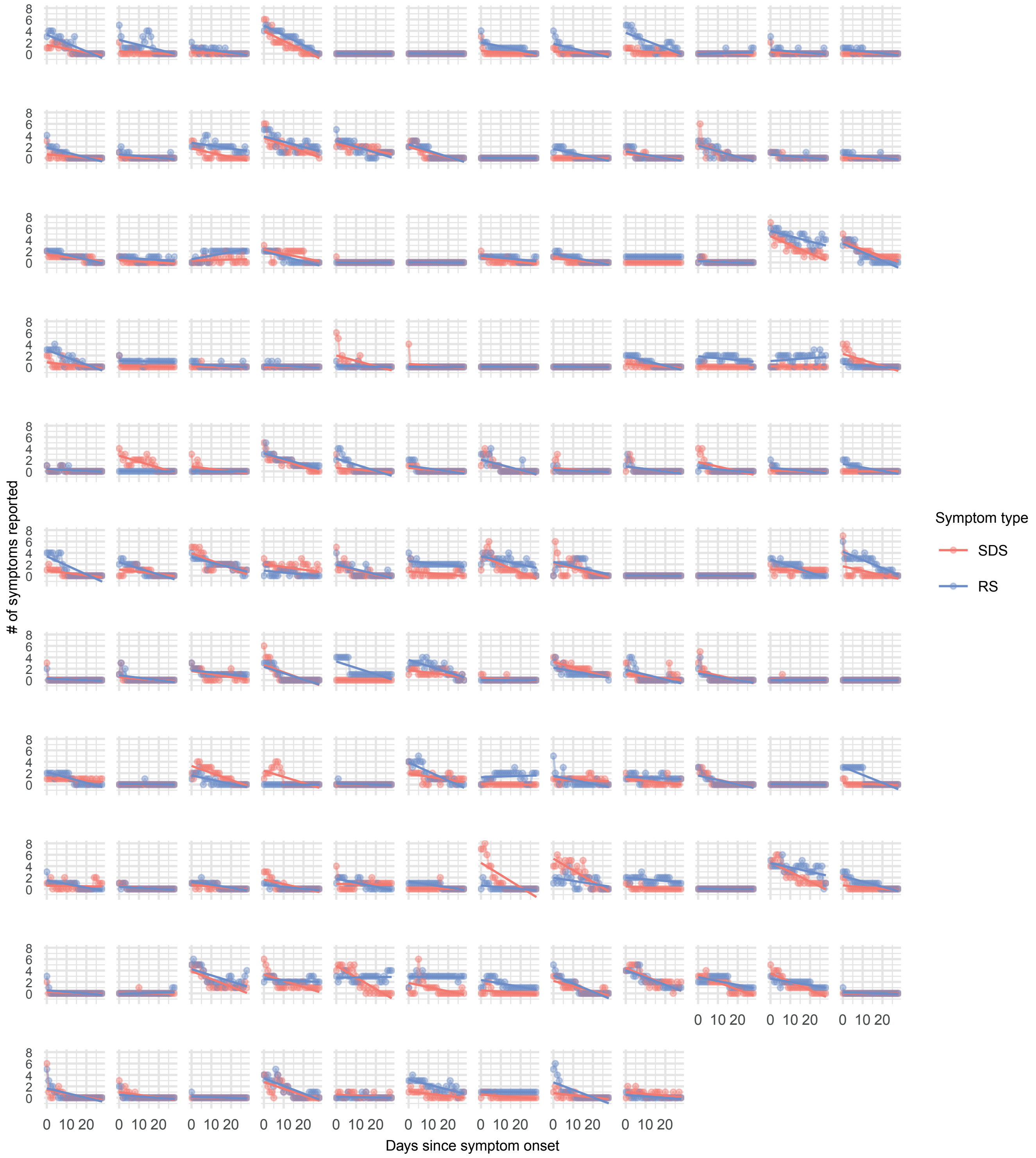

Figure S6. Individual longitudinal symptom data of cases. The number of respiratory (RS) and systemic disease (SDS) symptoms were determined for all cases (N = 130) for each day during the 28 day study period. Plotted are the raw symptom values per day as faded lines and datapoints, with the fitted values of the mixed-effects model as a solid line. The linear mixed-effect model was fit to the data per symptom group. The response was specified as the number of symptoms on a given day, and explanatory fixed effects variables were: Study day, age, average viral load on D0, and sex. Study day was also specified as a random slope, and Sample ID as a random intercept. Source data are provided as a Source Data file.

Variable	Total (n=187)	<18y (n=51)	18-49y (n=86)	50+y (n=50)
**Age** median [IQR]	34 [16-64]	11 [6.5-13.5]	34 [22-43]	55 [52-58]
**Female sex** n(%)	92 (49)	23 (45)	45 (52)	24 (48)
**Household members** n(%)	137 (73)	51 (100)	55(64)	31(62)
**Cases** n(%)	130 (70)	29 (57)	63 (73)	38 (76)
Among household members	*80 (58)*	*29 (57)*	*32 (58)*	*19 (61)*
*Positive at study start**	94 (72)	19 (66)	42 (67)	35 (92)
Seroconverted at day 28⁺	127 (98)	28 (97)	59 (94)	36 (95)
Nasoconverted at day 28⁺⁺	105 (81)	23 (79)	43 (68)	34 (89)

Table S1 | General characteristics of study participants, by age group. The PCR positive threshold was set at a Ct value <36, corresponding to a viral load of $\geq 10^3$ copies/ml of extracted sample. The seroconversion and nasoconversion threshold was based on the mean + 2*SD of the corresponding log2-transformed pre-SARS-CoV-2 control samples. *Participants who were PCR positive at day 0. ⁺Participants who showed seroconversion against the Spike protein at day 28. ⁺⁺ Participants who showed nasoconversion against the Spike protein at day 28.

	Index cases (n=50)		Household cases (n=80)		
	18-49y (N=31)	50+y (N=19)	<18y (N=29)	18-49y (N=32)	50+y (N=19)
**PCR +**	**27 (87)**	**19 (100)**	**19 (66)**	**15 (47)**	**14 (74)**
PCR+ sero+	*26*	*19*	*18*	*13*	*12*
PCR+ sero-	*1*	*0*	*1*	*2*	*2*
**Sero +**	**29 (94)**	**19 (100)**	**28 (97)**	**30 (94)**	**17 (89)**
PCR- sero+	*3*	*0*	*10*	*17*	*5*
**Naso +**	**28 (90)**	**18 (95)**	**23 (79)**	**15 (47)**	**16 (84)**
PCR+sero+naso+	*25*	*18*	*18*	*12*	*12*
PCR+sero-naso+	*0*	*0*	*0*	*0*	*1*
PCR-sero+naso+	*3*	*0*	*5*	*3*	*3*

Table S2 | PCR and serum positivity against SARS-CoV-2 in index and household cases. The PCR positive threshold was set at a Ct value <36, corresponding to a viral load of $\geq 10^3$ copies/ml of extracted sample. The seroconversion and nasoconversion threshold was based on the mean + 2*SD of the corresponding log2-transformed pre-SARS-CoV-2 control samples.

Table S3: primers used in qtPCR analysis.

Assay	Oligonucleotide	Sequence	Concentration*
E gene	E_Sarbeco_F	ACAGGTACGTTAATAGTTAATAGCGT	Use 400 nM per reaction
	E_Sarbeco_P1	FAM-ACACTAGCCATCCTTACTGCGCTTCG-BBQ	Use 200 nM per reaction
	E_Sarbeco_R	ATATTGCAGCAGTACGCACACA	Use 400 nM per reaction

* Optimised concentrations are given in nanomol per litre (nM) based on the final reaction mix, e.g. 1.5 µL of a 10 µM primer stock solution per 25 µL total reaction volume yields a final concentration of 600 nM as indicated in the table.

Source: https://doi.org/10.2807/1560-7917.ES.2020.25.3.2000045
