# Supplementary material for: SARS-CoV-2 mucosal antibody development and persistence and their relation to viral load and COVID-19 symptoms
Source: Nat Commun. 2021 Sep 23;12:5621. doi: 10.1038/s41467-021-25949-x (PMC8460778; doi:10.1038/s41467-021-25949-x)
Supplement: Supplementary file 3 — Reporting Summary [file 41467_2021_25949_MOESM3_ESM.pdf]

nature research

Corresponding author(s): Dimitri Diavatopoulos

Last updated by author(s): Aug 16, 2021

Reporting Summary

Nature Research wishes to improve the reproducibility of the work that we publish. This form provides structure for consistency and transparency in reporting. For further information on Nature Research policies, see our Editorial Policies and the Editorial Policy Checklist.

Statistics

For all statistical analyses, confirm that the following items are present in the figure legend, table legend, main text, or Methods section.

n/a	Confirmed	
☐	☒	The exact sample size (*n*) for each experimental group/condition, given as a discrete number and unit of measurement
☐	☒	A statement on whether measurements were taken from distinct samples or whether the same sample was measured repeatedly
☐	☒	The statistical test(s) used AND whether they are one- or two-sided<br>*Only common tests should be described solely by name; describe more complex techniques in the Methods section.*
☐	☒	A description of all covariates tested
☐	☒	A description of any assumptions or corrections, such as tests of normality and adjustment for multiple comparisons
☐	☒	A full description of the statistical parameters including central tendency (e.g. means) or other basic estimates (e.g. regression coefficient) AND variation (e.g. standard deviation) or associated estimates of uncertainty (e.g. confidence intervals)
☐	☒	For null hypothesis testing, the test statistic (e.g. *F*, *t*, *r*) with confidence intervals, effect sizes, degrees of freedom and *P* value noted<br>*Give P values as exact values whenever suitable.*
☒	☐	For Bayesian analysis, information on the choice of priors and Markov chain Monte Carlo settings
☒	☐	For hierarchical and complex designs, identification of the appropriate level for tests and full reporting of outcomes
☐	☒	Estimates of effect sizes (e.g. Cohen's *d*, Pearson's *r*), indicating how they were calculated

Our web collection on statistics for biologists contains articles on many of the points above.

Software and code

Policy information about availability of computer code

Data collection	Castor EDC (version 2020.1, clinical and demographical data), Luminex FlexMap3D System (Antibody data), Biorad CFX96 Real-Time PCR Detection System (version 3.1, viral load data)
Data analysis	Analysis of Luminex data was performed with Bio-Plex manager software (version 6.2.0.175, build 175, Bio-Rad Laboratories, Hercules, CA). Demographical data was exported from Castor EDC, and double checked with the paper records by two members of the research team. All statistical analyses were performed using the Rstudio environment (version 3.6.2), with libraries ‘stats’ (hypothesis tests and correlations), “lme4” , “lmerTest” for mixed-effects modelling and associated p-values, and “survival” for Kaplan-Meier survival analysis. The libraries “survminer” and “ggplot2” were used for visualization.

For manuscripts utilizing custom algorithms or software that are central to the research but not yet described in published literature, software must be made available to editors and reviewers. We strongly encourage code deposition in a community repository (e.g. GitHub). See the Nature Research guidelines for submitting code & software for further information.

Data

Policy information about availability of data

All manuscripts must include a data availability statement. This statement should provide the following information, where applicable:

- Accession codes, unique identifiers, or web links for publicly available datasets
- A list of figures that have associated raw data
- A description of any restrictions on data availability

The data that support the findings of this study are available from the corresponding author upon reasonable request.

Field-specific reporting

Please select the one below that is the best fit for your research. If you are not sure, read the appropriate sections before making your selection.

- [x] Life sciences
- [ ] Behavioural & social sciences
- [ ] Ecological, evolutionary & environmental sciences

For a reference copy of the document with all sections, see nature.com/documents/nr-reporting-summary-flat.pdf

Life sciences study design

All studies must disclose on these points even when the disclosure is negative.

Sample size	50 index cases were included, with at least two other participating household members per index case. This resulted in a total of 187 participants. There was no statistical sample size calculation done for this study, as there were no previous studies that could be used to obtain expected effect sizes. 50 index cases were chosen with an estimated total of 150 household members
Data exclusions	There were no individuals excluded from data analysis.
Replication	All PCR analyses were done in duplicate. MIA analyses were not completely done in replicate, but the measurements taken on day 28 were analysed twice to ascertain reproducibility of the assay. All MIA assays also included QC samples and the same reference curve, to enable batch effect detection and correction
Randomization	No randomization was preformed, as there was no intervention in this study
Blinding	No blinding was performed, as there was no intervention in this study

Reporting for specific materials, systems and methods

We require information from authors about some types of materials, experimental systems and methods used in many studies. Here, indicate whether each material, system or method listed is relevant to your study. If you are not sure if a list item applies to your research, read the appropriate section before selecting a response.

Materials & experimental systems

n/a	Involved in the study	
	x	Antibodies
x		Eukaryotic cell lines
x		Palaeontology and archaeology
x		Animals and other organisms
	x	Human research participants
	x	Clinical data
x		Dual use research of concern

Methods

n/a	Involved in the study	
x		ChIP-seq
x		Flow cytometry
x		MRI-based neuroimaging

Antibodies

Antibodies used	For detection in the multiplex immuno assay (MIA), the following antibodies were used: Goat anti-Human IgG-PE (Jackson Immunoresearch, 109-116-170), Goat anti-Human IgA-PE (Southern Biotech, 2052-09), and Goat anti-Human IgM-PE (Southern Biotech, 2022-09) were used in a dilution of 1:200. Specificity was checked using rabbit anti-SARS SIA-ST serum. No primary antibodies were used.
Validation	Assay validation had been performed in a previosuly published manuscript (https://doi.org/10.1093/infdis/jiaa479).

Human research participants

Policy information about studies involving human research participants

Population characteristics	Index cases were mostly female (76%) which is reflective of the gender amongst healthcare workers, with a median age of 46 (IQR: 37-54), while household members were mostly male (61%) and younger, with a median age of 21 (IQR:13-46).
Recruitment	Healthcare workers with a positive PCR for SARS-CoV-2 were contacted. Only those that were willing to participate and had had at least two other household members willing to participate were included. This could result in a bias of older people, as younger healthcare workers were more likely to not have two household members.
Ethics oversight	The study was approved by the medical ethics committee CMO Arnhem-Nijmegen, reference NL73418.091.20

Note that full information on the approval of the study protocol must also be provided in the manuscript.

Clinical data

Policy information about clinical studies
All manuscripts should comply with the ICMJEguidelines for publication of clinical research and a completedCONSORT checklist must be included with all submissions.

Clinical trial registration	NCT04590352
Study protocol	The full trial protocol can be sent upon request to the corresponding author. For the brief protocol, see clinicaltrials.gov
Data collection	Data was collected in the Netherlands, using three hospitals in the region Arnhem Nijmegen (CWZ, RadboudUMC and Rijnstate hospital) as the sources of the COVID-19 infected healthcare workers and their housemates. Registration was done using Castor EDC, in the period of 26th of March until the 15th of April 2020, with a follow-up measurement in December 2020 (9 months after study onset). Antibody data was generated by the Luminex FlexMap3D System and viral load data was generated with the CFX96 Real-Time PCR Detection System (BioRad)
Outcomes	Primary outcomes: Descriptive analysis of SARS-CoV-2 IgG, IgM and IgA concentrations in nasal fluid on day 0, 3, ,6, 7, 14, and 28. Done by performing a multiplex immuno-assay and analyzing the measured antibody intensities in R.<br><br>Secondary outcome measures: 1) Analysis of viral load, performed by a SARS-CoV-2 PCR on nasopharyngeal swab and throat swab. 2) Descriptive analysis of SARS-CoV-2 antibody concentrations in serum at day 28, performed by a multiplex immuno-assay and analyzing the measured antibody intensities in R. 3) Analysis of COVID-19 symptoms, done by categorizing the symptoms and looking at their resolution over time
